# AGE AND EMOTION: AGE MODERATES THE RELATIONSHIP BETWEEN MIXED EMOTIONAL EXPERIENCES AND PSYCHOLOGICAL WELL-BEING

**DOI:** 10.1093/geroni/igae098.4111

**Published:** 2024-12-31

**Authors:** Kathrine Whitman, Enna Chen, Jane Stephenson, Laura Carstensen

**Affiliations:** Stanford University, Palo Alto, California, United States; Stanford University, Palo Alto, California, United States; Stanford University, Palo Alto, California, United States; Stanford University, Palo Alto, California, United States

## Abstract

Aging is associated with higher levels of emotional well-being, with older adults experiencing more positive emotions, fewer negative emotions, and more mixed emotions in daily life. Mixed emotional experiences (reporting more than one type of emotion during the same period) may indicate more emotional complexity and adaptive functioning. Alternatively, it may indicate degraded emotional experience. Here, we examined the relationship between everyday positive, negative, and mixed emotional experiences with life satisfaction, trait-level positive emotion, psychological well-being, and depression. Participants in an experience sampling study (n =180, aged 18 to 93, M = 56.9) reported their emotions at five randomly selected times each day for seven days. As expected, reports of positive emotions were associated with life satisfaction and trait-level positive emotion, while negative emotions were associated with depression and inversely correlated with psychological well-being. Interestingly, the association of negative emotions with depression was weaker in older participants, suggesting that older people may be more resistant to clinical depression even in the face of negative emotions. Most interestingly, we found that mixed emotional states were associated with increased psychological well-being among older adults but decreased psychological well-being in younger adults. Older adults’ increased psychological well-being in relation to mixed emotions and their resilience to the effects of negative emotion are consistent with adaptive, age-related changes in emotional experience and well-being. These findings highlight the differential effects of experiential positive, negative, and mixed emotions on various measures of well-being, with younger and older adults experiencing key differences in emotional drivers of well-being.

